# Effect of acute and chronic liver diseases on the thyroid function in children

**DOI:** 10.1186/s12887-021-02816-8

**Published:** 2021-08-25

**Authors:** Gihan M. Bebars, Madeha A. Sayed, Lamia Hamdy, Reem A. Abdel Aziz

**Affiliations:** 1 Department of Pediatrics, Faculty of Medicine, Minia University, Minia, 61111 Egypt; 2 Department of Clinical Pathology, Faculty of Medicine, Minia University, Minia, Egypt

**Keywords:** Acute hepatitis, Chronic liver disease, Thyroid function tests

## Abstract

**Background:**

Thyroid hormones modulate hepatic function through regulation of basal metabolic rate in addition; the liver metabolizes the thyroid hormones and regulates their endocrine effects.

**Objectives:**

To assess thyroid functions in children with acute and chronic liver diseases.

**Methods:**

85 studied children were divided into 4 groups; group 1 (20 children) with acute hepatitis (AH), group 2 (20 children) chronic liver disease1 (CLD1; relatively preserved liver functions including Child-Pugh stage A), group 3 (20 children) chronic liver disease2 (CLD2; includes Child-Pugh stage B or C), group 4 (25 children) controls. All groups were subjected to detailed history, physical examination, Complete blood count, liver, renal function tests, viral markers, and thyroid functions (FT3, FT4, TSH).

**Results:**

Free T3 levels were lower in children with AH, CLD1 and CLD2. There was significant increase in TSH serum levels in CLD2.In acute hepatitis a negative correlation between serum free T4 and AST (r = -0.991), positive correlation between serum TSH and AST, VLDL, and cholesterol levels (r= 0.503, 0.533 and 0.498). A positive correlation between free T3 levels and prothrombin concentration (r= 0.991). Negative correlations between free T3 levels and PT, serum bilirubin and LDL serum levels in children with CLD2 (r= -0.992) (r= -0.902) and (r= -0.946)

**Conclusion:**

Acute and chronic liver diseases affect thyroid function in children and is correlated with the disease severity.

## What is known?

Euthyroid sick syndrome; defined as low fT3, normal or low fT4 and normal or low TSH and subclinical hypothyroidism (SH); defined as a serum TSH concentration above the reference range with normal serum fT4 and fT3 levels were documented in patients with chronic liver disease in adults.

## What is new?

- Very few studies which show the impact of the liver disease in children on the thyroid functions were conducted on children.
- The aim of our work is to study the effect of both acute and chronic liver disease on the thyroid functions in children.

## Background

Liver is essential for the synthesis of proteins and the metabolism of various hormones. Thus, liver diseases have been shown to be associated with various endocrinal disturbances [1]*.*

Unfortunately, recognition of liver disease in the pediatric patient remains a major problem. One factor is liver injury manifests in many ways; hence, different disorders have identical initial presentations [2]*.*

Liver plays an important role in the metabolism of thyroid hormones, as it is the most important organ in the peripheral conversion of tetraiodothyronine (T4) to T3 by Type 1 deiodinase conjugation, Moreover, it is involved in thyroid excretion, peripheral deiodination, and synthesis of thyroid-binding globulin (TBG) [3].

Serum concentrations of thyroid hormones vary in patients with hepatic disorders, especially liver cirrhosis [4].

The liver metabolizes thyroid hormones and regulates their systemic effects; therefore, liver diseases could affect thyroid hormone metabolism [5].

Thyroid hormones regulate the basal metabolic rate of all cells, including hepatocytes, and thereby modulate hepatic function; the liver in turn metabolizes the thyroid hormones and regulates their endocrine effects. Thyroid dysfunction may affect liver functions, also liver disease modulates thyroid hormone metabolism [6]*.*

The aim of our study is to assess thyroid functions in children with acute and chronic liver diseases in order that we could detect the impact of the liver diseases on the thyroid functions.

## Methods

### Patients

This is a prospective case control comparative study, conducted on 60 children with liver diseases and 25 age and sex matched apparently healthy children as controls. Our patients were collected from the hepatology outpatient clinic and from the inpatient unit of gastroenterology and hepatology unit of Minia Children University Hospital. Controls were collected from outpatient general clinic of the same hospital over the period from September 2019 to September 2020.

Our patients were divided into 4 groups:

Group 1: Included 20 children with acute hepatitis (AH) diagnosed based on acute onset of jaundice and a threefold rise in serum transaminases [1].

Group 2: Included 20 children with chronic liver disease1 (CLD1).

Group 3: Included 20 children with chronic liver disease2 (CLD2)

Both group 2 (chronic liver disease 1) and group 3 (chronic liver disease 2) were classified according to child - pugh stage score [7].

Group 4: Included 25 age and sex matched apparently healthy children as controls.

Child - pugh stage score [7]

**Table:** 

	**1**	**2**	**3**
**Encephalopathy**	None	Grade 1-2 (precipitant-induced)	Grade 3-4 (chronic)
**Ascites**	None	Mild/Moderate (diuretic-responsive)	Severe (diuretic-refractory)
**Bilirubin (mg/dL)**	<2	2-3	>3
**Albumin (g/dL)**	>3.5	2.8-3.5	<2.8
**PT (sec prolonged)** **or INR**	<4 <1.7	4-6 1.7-2.3	>6 >2.3

*PT* prothrombin time, *INR* international normalized ratio

Total score of 5-6= stage A (well compensated disease)

Total score of 7-9= stage B (disease with significant functional compromise)

Total score of 10-15= stage C (decompensated liver disease) [5]

- Chronic liver disease1 (CLD1) with relatively preserved liver functions includes Child-Pugh stage A (Group2)

- Chronic liver disease2 (CLD2) includes Child-Pugh stage B or C (Group3)

Children with acute hepatitis (acute onset of jaundice and three-fold rise in serum transaminases) and chronic liver disease (evidence of liver disease of more than 6 months duration and/or portal hypertension on ultrasonography or upper gastrointestinal endoscopy) were included in the study

Patients on steroids, thyroid hormone therapy, medications that can affect metabolism of thyroid hormone e.g Interferon, Interleukin-2, Amiodarone, Dopamine agonists, Somatostatin analogs, chemotherapy and patients who had non-alcoholic steatohepatitis, cardiac, pancreatic diseases or malignancy were excluded from our study.

## Methods

Studied children were subjected to thorough history taking, age, sex, history of drug intake, previous operations, or blood transfusion. general examination; vital signs, conscious level, and anthropometric measures (weight, length/ height, head circumference and BMI), clubbing, pain, tenderness, edema and bleeding tendency. Skin examination; for jaundice, pallor, subcutaneous hemorrhage, and spider nevi. Systemic examination included abdominal examination; hepatomegaly, splenomegaly, ascites and its degree if mild, moderate or tense ascites. Cardiovascular and respiratory examination for complications as pneumonia and bronchiectasis was also done. Both verbal and written consents were asked from all patients’ guardians.

### Laboratory investigations

#### Blood sampling

Under complete aseptic conditions, 4 ml venous blood samples were withdrawn from each patient; one ml venous blood was collected into a tube containing ethylene- diamine-tetra acetic acid (EDTA) for complete blood count, another one ml was collected into a tube containing tri-sodium citrate for determination of prothrombin time and concentration. Finally, 2 ml venous blood were withdrawn into a plane tube, left to clot, centrifuged and stored at -70°C for determination of liver, renal function tests, viral markers and thyroid functions (FT3, FT4, TSH).

Complete blood count: It was determined by automated cell counter, Sysmex K-800, TAO Medical Incorporation, Japan.

Chemical analyses: lipid profile, serum ammonia, liver and renal function tests were done by using auto analyzer Kone-lab (20 I), Thermo electron, Clinical chemistry automation systems, Finland.

Prothrombin time and concentration were done by using option 2, Vitek, Inc .595, USA.

Enzyme Immunoassay (EIA) test was used for quantitative determination of free tri-iodothyronine hormone, free thyroxine hormone and thyroid stimulating hormone (TSH) in human serum kit supplied by Chemux Bioscience, Inc (BIOS), USA [8].

Normal free T3 level: 2.5- 5.0 Pg/ml

Normal free T4 level: 0.97- 1.67 ng/dl

Normal TSH level: 0.6- 4.84 mIU/ml [9]

Abdominal ultrasonography was done for all our patients to detect hepatomegaly, splenomegaly, ascites, and portal hypertension.

### Statistical methods

Data entry and analysis were all done with I.B.M. compatible computer using software called SPSS for windows version 13. Graphics were done by Excel.

Quantitative data were presented by mean and standard deviation, while qualitative data were presented by frequency distribution. Chi square test, fisher exact test was used to compare between proportions. One-way ANOVA and Student t-test was used to compare two means. Spearman correlation test was used.

The probability of less than 0.05 was used as a cut off point for all significant tests.

## Results

This is a prospective case control comparative study included 60 patients and 25 controls.

There was no statistical difference between AH, CLD1 and CLD2 regarding age, sex, clinical presentation except for melena and ascites in CLD2 (Table 1).

**Table 1:** Demographic and clinical data of the studied groups.

		Group I	Group II	Group III	Group IV	P value
		Acute hepatitis	CLD1	CLD2	Control	
		N=20	N=20	N=20	N=25	
**Age**	Range Mean ± SD	(2.5-8) 5.5±1.5	(3-6) 4.6±1	(3-7) 5±1.3	(3-7) 5±1	0.117
**Sex**	Male Female	8(40%) 12(60%)	14(70%) 6(30%)	10(50%) 10(50%)	14(56%) 11(44%)	0.283
**Heamatemsis**	+ve -ve	2 (10%) 18(90%)	4(20%) 16(80%)	7(35%) 13(65%)		0.1
**Melena**	+ve -ve	2(10%) 18(90%)	10(50%) 10(50%)	15(75%) 5(25%)		0.001*
**Jaundice**	+ve -ve	20(100%) 0	16(80%) 4(20%)	17(85%) 3(15%)		0.1
**Hepatomegaly**	+ve -ve	2(10%) 18(90%)	12(60%) 8(40%)	14(70%) 6(30%)		0.4
**Splenomegaly**	+ve -ve	2(10%) 18(90%)	12(60%) 8(40%)	15(75%) 5(25%)		0.1
**Ascitis**	+ve -ve	0 20(100%)	8(40%) 12(60%)	10(50%) 10(50%)		0.001*
**Edema**	+ve -ve	0 20(100%)	4(20%) 16(80%)	10(50%) 10(50%)		0.3
**Hepatic encephalopathy**	+ve -ve	0 20(100%)	4(20%) 16(80%)	2(10%) 18(90%)		0.1

One Way ANOVA test for parametric quantitative data between the four groups followed by post hoc Tukey analysis between each two groups

Chi square test for qualitative data between groups

* significant *p* value

Portal hypertension was detected in 11 children with CLD2 and only in one case with CLD1.

Hb levels were decreased in all affected children in comparison to the control group. Serum Na and K were significantly decreased in CLD2 (Table 2)

**Table 2:** CBC and Serum Electrolytes of the studied groups

		Group I	Group II	Group III	Group IV	P value
		Acute hepatitis	CLD1	CLD2	Control	
		N=20	N=20	N=20	N=25	
**Hb**	*Range* *Mean ± SD*	(9-9.7) 9.3±0.3	(9-9.6) 9.2±0.2	(9-9.8) 9.3±0.3	(9.8-14.8) 12.1±1.1	<0.001*
**WBCs**	*Range* *Mean ± SD*	(4-7) 5.6±0.9	(4-7) 5.2±0.9	(4-7) 5.9±0.9	(2.8-6.5) 5.3±0.9	*0.069*
**RBCs**	*Range* *Mean ± SD*	(4-9) 4.9±1	(4.7-5) 4.8±0.1	(4.7-5) 4.9±0.1	(4-5.6) 4.9±0.4	*0.946*
**PLT**	*Range* *Mean ± SD*	(256-300) 272.7±16.3	(268-300) 284.7±10.9	(250-300) 280.3±17	(140.5-423.3) 293.4±62.1	*0.284*
**S Na**	*Range* *Mean ± SD*	(130-140) 136±3.8	(130-140) 136.2±2.9	(120-124) 122.5±1.3	(129.9-143.6) 137.6±3.2	<0.001*
**Serum K**	*Range* *Mean ± SD*	(3.5-5) 3.9±0.4	(3.7-5) 4.2±0.5	(1.6-2.3) 1.9±0.2	(3.3-4.1) 3.8±0.2	<0.001*

* significant *p* value

Children with acute hepatitis had significant elevation of liver enzymes, serum total and direct bilirubin, while children with CLD2 showed significant elevation in blood urea, creatinine, serum ammonia, and lactate with prolonged Prothrombin time and low prothrombin concentration. Moreover, a high lipid profile in affected children than control. (Table 3)

**Table 3:** Liver, Renal functions, Serum Ammonia, Lactate and Lipid profile of the studied groups

		Group I	Group II	Group III	Group IV	P value
		Acute hepatitis	CLD1	CLD2	Control	
		N=20	N=20	N=20	N=25	
**ALT**	*Range* *Mean ± SD*	(700-900) 812±75	(200-350) 290±44.7	(100-200) 163±32.8	(16.7-43.3) 29.8±7.2	<0.001*
**AST**	*Range* *Mean ± SD*	(500-700) 609.5±85.9	(150-200) 169±21.7	(100-150) 134±13.9	(10.7-50.2) 32.3±9.2	<0.001*
**T. Billirubin**	*Range* *Mean ± SD*	(10-12) 11.3±0.6	(5-6.8) 5.8±0.7	(3-5) 4.3±0.6	(0.2-0.9) 0.6±0.2	<0.001*
**D. Billirubin**	*Range* *Mean ± SD*	(5-8) 6.5±1	(3-4) 3.6±0.4	(2-3) 2.7±0.2	(0.1-0.1) 0.1±0	<0.001*
**Bl Urea**	*Range* *Mean ± SD*	(15-20) 17.4±1.6	(22-30) 27.1±2.7	(34-40) 37.8±1.8	(10.5-19.7) 15.4±2.7	<0.001*
**S.Creatinin**	*Range* *Mean ± SD*	(0.5-0.7) 0.5±0.1	(0.5-0.6) 0.5±0.1	(0.7-0.9) 0.8±0.1	(0.4-0.7) 0.5±0.1	<0.001*
**PT**	*Range* *Mean ± SD*	(11-13) 11.8±0.7	(13-16) 14.6±1.2	(15-20) 17.3±1.8	(9.1-11.6) 10.1±0.6	<0.001*
**PC**	*Range* *Mean ± SD*	(97-100) 98.5±0.9	(75-80) 77.2±2.2	(40-50) 44.9±3.4	(95.4-101.6) 99±1.6	<0.001*
**S Ammonia**	*Median* *IQR*	14 (13-15)	55 (55-60)	98 (95-100)	9.7 (7.4-17.7)	<0.001*
**S Lactate**	*Median* *IQR*	1.5 (1-2)	5 (4.5-6)	26 (24-27)	2.6 (1-4.4)	<0.001*
**LDL**	*Range* *Mean ± SD*	(103-115) 110.1±4.5	(110-120) 115.8±3.9	(180-190) 186.2±2.8	(86.4-108.6) 99.6±6.1	<0.001*
**VLDL**	*Range* *Mean ± SD*	(30-35) 33.4±1.8	(30-35) 32.9±1.9	(35-53) 39.6±5.2	(20.2-44.1) 29.6±5.3	<0.001*
**HDL**	*Range* *Mean ± SD*	(30-35) 33.5±1.8	(30-35) 33±1.8	(30-35) 33.4±1.8	(46.3-67.4) 54.9±5.2	<0.001*
**Cholesterol**	*Range* *Mean ± SD*	(140-160) 150.2±7	(180-190) 186±3.8	(180-195) 186.7±5	(111.1-219.7) 150.4±26.1	<0.001*
**Triglyceride**	*Range* *Mean ± SD*	(115-120) 117.3±2	(180-190) 184.7±3.7	(180-190) 186.6±3.5	(94-140.4) 121.3±11.9	<0.001*

One Way ANOVA test for parametric quantitative data between the four groups followed by post hoc Tukey analysis between each two groups

*: Significant level at *P* value < 0.05

**Fig. 1:**
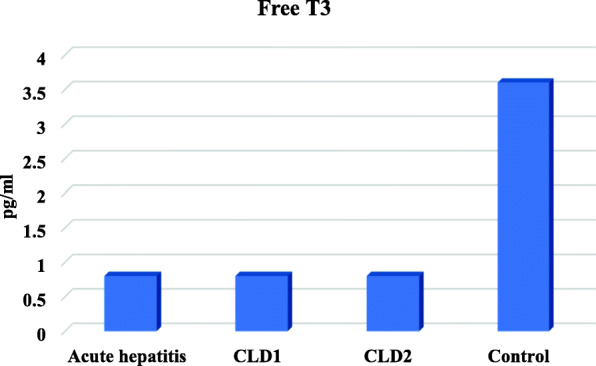
Serum FT3 levels in the studied groups

**Fig. 2:**
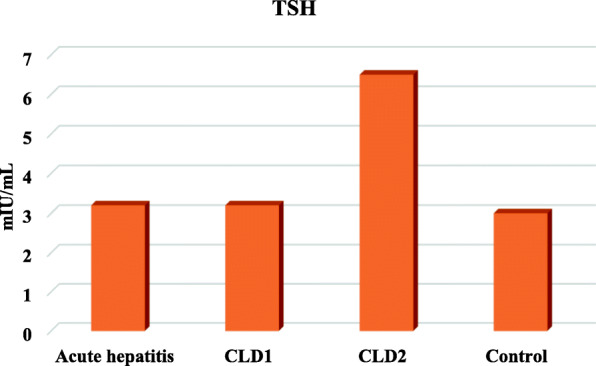
Serum TSH levels in the studied groups

**Fig. 3:**
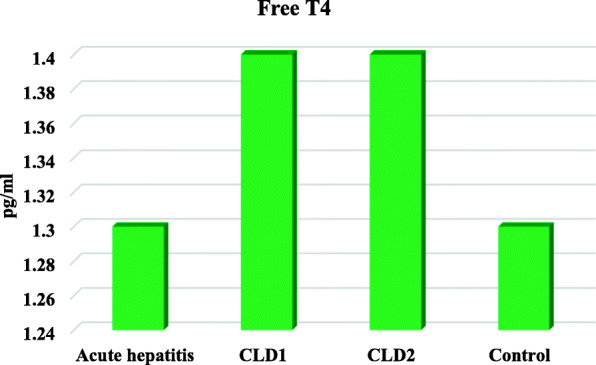
Serum FT4 levels in the studied groups

Free T3 levels were lower in children with AH, CLD1 and CLD2 than controls but, there were significant increase in TSH serum levels only in children with CLD2 (Table 4, Figs. 1, 2 and 3)

**Table 4:** Free T3, T4 and TSH in the studied groups

		Group I	Group II	Group III	Group IV	P value
		Acute hepatitis	CLD1	CLD2	Control	
		N=20	N=20	N=20	N=25	
**Free T3**	*Range* *Mean ± SD*	(0.5-1.2) 0.8±0.2	(0.5-1.2) 0.8±0.2	(0.5-1) 0.8±0.2	(2.6-4.9) 3.6±0.6	<0.001*
**Free T4**	*Range* *Mean ± SD*	(0.9-1.7) 1.3±0.4	(1-1.7) 1.4±0.2	(1-1.7) 1.4±0.2	(1-1.7) 1.3±0.2	*0.397*
**TSH**	*Range* *Mean ± SD*	(1.2-4.9) 3.2±1.1	(1.9-4.8) 3.2±0.9	(6.2-6.9) 6.5±0.2	(0.8-4.8) 3±1.2	<0.001*

One Way ANOVA test for parametric quantitative data between the four groups followed by post hoc Tukey analysis between each two groups

*: Significant level at *P* value < 0.05

In patients with acute hepatitis, there was negative correlation between serum free T4 levels and AST (r = -0.991 & p< 0.001), positive correlation between serum TSH levels and AST, VLDL, and cholesterol levels (r= 0.503, 0.533 and 0.498 & p= 0.024, 0.015 and 0.026 respectively) (Table 5)

**Table 5:** Correlations between FT3, FT4, FSH and Laboratory data of Group I (Acute Hepatitis)

Acute Hepatitis group	Free T3		Free T4		TSH	
	r	P value	r	P value	r	P value
**Age**	*0.104*	*0.663*	*0.299*	*0.201*	*-0.356*	*0.124*
**Hb**	*-0.158*	*0.507*	*-0.090*	*0.705*	*-0.109*	*0.648*
**WBCs**	*0.340*	*0.143*	*-0.145*	*0.543*	*0.040*	*0.866*
**RBCs**	*0.006*	*0.981*	*-0.143*	*0.547*	*0.403*	*0.078*
**PLT**	*0.317*	*0.174*	*-0.176*	*0.458*	*0.347*	*0.134*
**ALT**	*0.209*	*0.378*	*-0.073*	*0.758*	*0.130*	*0.584*
**AST**	*-0.173*	*0.466*	*-0.991*	<0.001*	*0.503*	0.024*
**T. Bilirubin**	*0.053*	*0.825*	*-0.061*	*0.799*	*0.348*	*0.133*
**D. Bilirubin**	*0.089*	*0.710*	*-0.397*	*0.083*	*0.084*	*0.725*
**Bl Urea**	*0.235*	*0.319*	*-0.270*	*0.249*	*0.280*	*0.232*
**S. Creatinine**	*0.054*	*0.822*	*-0.193*	*0.415*	*0.190*	*0.422*
**PT**	*0.129*	*0.588*	*0.025*	*0.917*	*-0.075*	*0.754*
**PC**	*-0.267*	*0.255*	*0.159*	*0.502*	*0.242*	*0.304*
**LDL**	*-0.038*	*0.872*	*-0.222*	*0.347*	*0.410*	*0.073*
**VLDL**	*0.134*	*0.574*	*-0.339*	*0.144*	*0.533*	0.015*
**HDL**	*0.300*	*0.198*	*0.214*	*0.365*	*0.102*	*0.669*
**Cholesterol**	*0.059*	*0.804*	*-0.225*	*0.339*	*0.498*	0.026*
**Triglyceride**	*-0.103*	*0.665*	*-0.176*	*0.459*	*0.156*	*0.511*
**S Ammonia**	*0.402*	*0.079*	*0.004*	*0.986*	*-0.066*	*0.781*
**S Lactate**	*-0.050*	*0.833*	*0.163*	*0.492*	*0.074*	*0.757*
**S Na**	*-0.197*	*0.405*	*0.086*	*0.717*	*0.088*	*0.712*
**Serum K**	*-0.064*	*0.787*	*-0.105*	*0.658*	*0.043*	*0.858*

Pearson’s correlation

*: Significant level at *P* value < 0.05

There was no significant correlation between thyroid functions and any laboratory data in patients with CLD1 (Table 6)

**Table 6:** Correlations between FT3, FT4, FSH and Laboratory data of Group II (CLD1)

CLD1 group	Free T3		Free T4		TSH	
	r	P value	r	P value	r	P value
**Age**	*-0.415*	*0.069*	*0.051*	*0.830*	*-0.092*	*0.699*
**Hb**	*-0.260*	*0.269*	*0.230*	*0.329*	*0.215*	*0.362*
**WBCs**	*0.011*	*0.964*	*0.263*	*0.117*	*-0.174*	*0.464*
**RBCs**	*0.205*	*0.386*	*-0.181*	*0.445*	*0.399*	*0.082*
**PLT**	*0.177*	*0.456*	*0.154*	*0.516*	*0.144*	*0.545*
**ALT**	*0.206*	*0.384*	*0.192*	*0.403*	*0.277*	*0.237*
**AST**	*0.042*	*0.859*	*-0.214*	*0.265*	*-0.156*	*0.511*
**T. Bilirubin**	*0.304*	*0.219*	*-0.139*	*0.559*	*0.133*	*0.576*
**D. Bilirubin**	*0.184*	*0.438*	*0.156*	*0.248*	*-0.186*	*0.431*
**Bl Urea**	*0.075*	*0.754*	*-0.066*	*0.782*	*0.311*	*0.182*
**S. Creatinine**	*-0.110*	*0.644*	*0.194*	*0.414*	*-0.404*	*0.078*
**PT**	*0.338*	*0.145*	*-0.292*	*0.212*	*0.186*	*0.432*
**PC**	*-0.190*	*0.421*	*-0.012*	*0.959*	*0.236*	*0.316*
**LDL**	*0.341*	*0.141*	*0.182*	*0.442*	*0.107*	*0.653*
**VLDL**	*-0.154*	*0.517*	*0.236*	*0.317*	*0.236*	*0.379*
**HDL**	*-0.211*	*0.372*	*-0.050*	*0.834*	*0.076*	*0.751*
**Cholesterol**	*-0.050*	*0.833*	*0.144*	*0.545*	*0.205*	*0.385*
**Triglyceride**	*-0.057*	*0.812*	*0.207*	*0.259*	*0.212*	*0.370*
**S Ammonia**	*0.103*	*0.667*	*-0.036*	*0.880*	*0.321*	*0.142*
**S Lactate**	*0.145*	*0.541*	*0.151*	*0.525*	*0.207*	*0.381*
**S Na**	*-0.030*	*0.898*	*0.345*	*0.136*	*0.268*	*0.534*
**Serum K**	*-0.028*	*0.907*	*0.111*	*0.643*	*0.106*	*0.656*

Pearson’s correlation

*: Significant level at *P* value < 0.05

A positive correlation between free T3 levels and prothrombin concentration (r= 0.991, P< 0.001). Negative correlations between free T3 levels and PT, serum bilirubin and LDL serum levels in children with CLD2 (r= -0.992, P = 0.001) (r= -0.902, P= 0.001) and (r= -0.946, p= 0.001) respectively (Table 7).

**Table 7:** Correlations between FT3, FT4, FSH and Laboratory data of Group III (CLD2)

CLD2 group	Free T3		Free T4		TSH	
	r	P value	r	P value	r	P value
**Age**	*-0.252*	*0.284*	*-0.118*	*0.622*	*0.038*	*0.875*
**Hb**	*0.135*	*0.569*	*-0.276*	*0.240*	*0.027*	*0.909*
**WBCs**	*-0.030*	*0.899*	*-0.302*	*0.195*	*-0.394*	*0.086*
**RBCs**	*0.141*	*0.554*	*-0.241*	*0.305*	*-0.144*	*0.544*
**PLT**	*-0.317*	*0.173*	*-0.067*	*0.779*	*0.398*	*0.082*
**ALT**	*-0.317*	*0.173*	*0.170*	*0.475*	*0.080*	*0.736*
**AST**	*-0.183*	*0.441*	*0.301*	*0.197*	*0.180*	*0.447*
**T. Bilirubin**	*-0.902*	<0.001*	*0.295*	*0.207*	*0.004*	*0.986*
**D. Bilirubin**	*-0.333*	*0.152*	*0.369*	*0.109*	*-0.119*	*0.616*
**Bl Urea**	*-0.367*	*0.111*	*-0.071*	*0.767*	*-0.119*	*0.617*
**S. Creatinine**	*-0.326*	*0.160*	*0.134*	*0.573*	*0.045*	*0.851*
**PT**	*-0.992*	<0.001*	*0.335*	*0.148*	*-0.017*	*0.942*
**PC**	*0.991*	<0.001*	*-0.319*	*0.170*	*0.049*	*0.839*
**LDL**	*-0.946*	<0.001*	*0.352*	*0.128*	*-0.109*	*0.647*
**VLDL**	*-0.101*	*0.673*	*0.193*	*0.414*	*0.004*	*0.988*
**HDL**	*0.316*	*0.175*	*0.201*	*0.396*	*-0.041*	*0.863*
**Cholesterol**	*-0.056*	*0.813*	*0.185*	*0.436*	*-0.308*	*0.186*
**Triglyceride**	*0.011*	*0.964*	*0.026*	*0.913*	*0.126*	*0.597*
**S Ammonia**	*0.117*	*0.624*	*0.104*	*0.662*	*-0.281*	*0.230*
**S Lactate**	*-0.218*	*0.355*	*0.147*	*0.535*	*0.059*	*0.804*
**S Na**	*-0.209*	*0.377*	*0.134*	*0.574*	*-0.052*	*0.827*
**Serum K**	*0.083*	*0.729*	*0.089*	*0.709*	*-0.036*	*0.879*

Pearson’s correlation

*: Significant level at *P* value < 0.05

## Discussion

Endocrinal disturbances are common with liver diseases which are correlated with severity of liver disease [10].

The liver is essential for thyroid hormones metabolism, and it regulates their systemic effects. At the same time, a healthy thyroid is essential for a good healthy liver [8].

To the best of our knowledge, this is one of the few studies conducted on children to assess thyroid functions with both acute and chronic liver disease using Child-Pugh stage score. Many studies assessing thyroid function tests in liver disease conducted on adults. We have conducted this study on twenty children with acute hepatitis (AH), 20 children with chronic liver disease1 (CLD1), and 20 children with chronic liver disease2 (CLD2).

No differences between the three studied groups regarding age, sex or anthropometric measures. and this agreed with the study of *Sandeep, 2015* [11].

There was no significant difference among the studied groups with different liver diseases regarding the frequency of presentation for hepatomegaly, splenomegaly, or jaundice, but a significant increase in the rates of ascites, melena, and portal hypertension in children with CLD2. these results were partially in accordance with Steven Kim, 2015 [12].

A significant elevation in portal pressure in CLD2 group which was in concordance with the study of Sandeep, 2015 who found significant elevation of portal pressure in cirrhotic patients that is explained by periportal fibrosis [11].

Despite the liver’s tremendous capacity for regeneration, chronic liver injury can result in fibrosis and eventually end-stage liver disease. Classically, aminotransferase elevation is interpreted as a marker of hepatocellular damage [13]. This was also detected in the current study as the three groups with liver disease showed a significant elevation in liver enzymes levels (ALT & AST).

In contrast to our study, another study included patients with chronic hepatitis B infection showed normal ALT, defined as less than 40 U/L, found histological disease activity in 14% to 40%, depending on e antigen status [14]. Also, a study by Hsu and Murray, 2014 reported no significant difference between children with liver cirrhosis regarding hepatic enzymes [15].

Our study detected a significant elevation in both serum ammonia and lactate levels in children with CLD2. These findings were in agreement with the study of Walther’s study, 2013 who found the same significant differences in children with hepatocellular carcinoma [16].

High ammonia levels may be a sign of Liver diseases, such as hepatitis, cirrhosis, or hepatic encephalopathy [17].

HAV - Ab were detected in 15 (75%) of children with acute hepatitis and 4 (20%) of CLD1. HBsAg was positive in 4 (20%) of children with CLD1 and 1 (5%) in CLD2 while HCV-Ab was positive in 8 (40%) in children with CLD1 and 11 (55%) in CLD2. No cases had mixed infections with hepatitis viruses (P= 0.001).

These findings agreed with study of Sandeep, 2015 as 18 patients with acute hepatitis were (HAV-Ab) +ve and among 50 adults with CLD, 29 were hepatitis B virus (HBV) related cirrhosis, 8 were hepatitis C virus (HCV) related cirrhosis, and 13 had HBV and HCV coinfection [11].

A significant decrease in free T3 levels in all children with AH, CLD1, and CLD2 in comparison to controls were detected in the study. This decrease in FT3 also documented in the study of Sandeep, 2015 [11]*.* In several studies, low FT3 levels were the most consistent finding. In Deepika *et al*., D’costa and Dhume, Saleem and Wadea, El-Sawy and Tawfi, the levels of FT3 were significantly low in patients with liver cirrhosis [18–21].

Regarding serum free T4 levels, there was no statistical difference in all patients’ groups in comparison to controls. This was in agreement with study of Punekar et al, 2018 found a significant decrease in free T3 levels but insignificant decrease in free T4 [6].

Kayacetin *et al, 2003* reported that serum levels of FT3 and total T4 were significantly lower in all cirrhotic patients [22].

But, the study of Sandeep, 2015 found significant increased T4 in patients with CLD1 and decreased T4 in patients with CLD2 [11].

In our study, there was significant increase in TSH levels only in children with CLD2.

Mobin *et al*., 2016, El-Feki et al., 2016 and Punekar et al, 2018, reported that all decompensated cirrhotic patients had low serum T3 levels, serum T4 levels, and raised TSH levels [6, 23, 24].

So, the results of our study for FT3 levels and TSH levels are consistent with Mobin *et al* study but contradict for FT4 levels. This difference may be due to severity of liver disease and regional variation of thyroid disorders.

Our result is in contrast with study of Sandeep, (2015), *who* reported decrease in level of TSH; this reduction of TSH level may be due to liver disease that is associated with increase in inflammatory cytokines that negatively affect hypothalamo-thyroid axis [11].

Euthyroid sick syndrome; defined as low fT3, normal/low fT4 and normal/low TSH were documented in Catli et al study [25] and subclinical hypothyroidism (SH); defined as a serum TSH concentration above the reference range with normal serum fT4 and fT3 levels in de Vries study and Ön et al. [26, 27].

There are many factors may be responsible for these abnormalities, which includes alteration in plasma level of thyroid binding proteins, altered binding of T4 and T3 to their carrier protein, impaired hepatic clearance of reverse T3, hyperglucagonemia, and reduced extrathyroidal conversion of T4 to T3. In cirrhotic patients, because of extensive hepatic inflammation and fibrosis, there is inhibition of Type 1 (D1) deiodinase enzymes that lead to decreased conversion of T4 to T3 [6].

In children with acute hepatitis, free T4 levels were negatively correlated with AST levels (r= -0.991, P<0.001) where TSH levels were positively correlated with AST, VLDL and cholesterol levels (r= 0.503, 0.533, 0.498, P= 0.024, 0.015, 0.026 respectively). There was no significant correlation between thyroid functions and any laboratory data in patients with CLD1.

But the study of *Ayub, 2010* found no significant correlation between thyroid hormones and liver enzymes [28].

On contrast, there was positive correlation between free T3 levels and prothrombin concentration (r= 0.991, P< 0.001). Negative correlations between serum T3 levels and PT, serum bilirubin and LDL serum levels in children with CLD2 (r= -0.992, P = 0.001) (r= -0.902, P= 0.001) and (r= -0.946, p= 0.001) respectively in studied children with CLD2.

These findings were partially in agreement with study of Malik and Hodgson*,* 2002 who conducted his study on 118 patients with acute and chronic liver disease to evaluate the relationship between thyroid gland and liver diseases, they found negative correlation between T4 and liver enzymes in children with acute and chronic liver diseases, and no significant correlation between T3 and PT, PC & serum bilirubin in children with chronic liver diseases [29].

Sandeep, 2015 reported a negative correlation between LDL and T3 levels in children with CLD2 and was explained as Thyroid hormones increase the expression of LDL receptors on the hepatocytes and increase the activity of lipid-lowering liver enzymes, resulting in a reduction in low-density lipoprotein levels. Thyroid hormones also increase the expression of apolipoprotein A1. Hence, decrease in thyroid hormones associated with liver disease will adversely affect LDL disposal and decrease HDL synthesis [11].

Punekar et al., 2018 stated that in all cirrhotic patients, FT3 and FT4 were negatively correlated, but TSH level was positively correlated with total leukocyte counts, serum total bilirubin, aspartate transaminase, alanine transaminase, prothrombin time (PT), blood urea and serum creatinine. They concluded that level of FT3, FT4, and TSH also correlate with the severity of liver disease. Level of FT3 can be used as prognostic marker for liver cirrhosis patients [6].

Ön etal., 2020 found that there was a negative correlation between fT3 and direct bilirubin (r=-0.329, p=0.027). They also stated that there was a negative correlation between fT4 and total albumin (r=-0.273, p=0.005) [27]

## Conclusion

Free T3 levels were significantly lower in children with acute hepatitis. TSH serum levels significantly increased in children with CLD2. Serum free T4 levels were negatively correlated to AST. positive correlation between serum TSH levels and AST, VLDL, and cholesterol levels in patients with acute hepatitis.

Free T3 levels are correlated with the disease severity as they are positively correlated with prothrombin concentration, negatively correlated to PT, serum bilirubin and LDL serum levels in children with CLD2.

### Impact on society

Thyroid function test should be done regularly in all patients with liver disease in order to reduce morbidity and mortality.

## Data Availability

All datasets used and/or analyzed during the current study are available from the corresponding author on reasonable request.

## Abbreviations

AH: Acute hepatitis
ALT: Alanine aminotransferase
AST: Aspartate aminotransferase
BMI: Body mass index
CLD: Chronic liver disease
EDTA: Ethylene- diamine-tetra acetic acid
EIA: Enzyme Immunoassay
FT3: Free triodothyronine
FT4: Free tetraiodothyronine
HAV-Ab: Hepatitis A antibody
HBsAg: Hepatitis B surface antigen
HCV-Ab: Hepatitis C antibody
INR: International normalized ratio
LDL: Low density lipoprotein
PT: Prothrombin time
T4: Tetraiodothyronine
TBG: Thyroid-binding globulin
TSH: Thyroid Stimulating Hormone
VLDL: Very low-density lipoprotein
